# Impact of intraoperative magnetic resonance imaging on gross total resection, extent of resection, and residual tumor volume in pituitary surgery: systematic review and meta-analysis

**DOI:** 10.1007/s11102-021-01147-2

**Published:** 2021-05-04

**Authors:** Victor E. Staartjes, Alex Togni-Pogliorini, Vittorio Stumpo, Carlo Serra, Luca Regli

**Affiliations:** Machine Intelligence in Clinical Neuroscience (MICN) Laboratory, Department of Neurosurgery, Clinical Neuroscience Center, University Hospital Zurich, University of Zurich, Frauenklinikstrasse 10, 8091 Zurich, Switzerland

**Keywords:** Pituitary, Adenoma, Intraoperative magnetic resonance imaging, Imaging, Extent of resection, Gross total resection

## Abstract

**Background:**

Residual tumor tissue after pituitary adenoma surgery, is linked with additional morbidity and mortality. Intraoperative magnetic resonance imaging (ioMRI) could improve resection. We aim to assess the improvement in gross total resection (GTR), extent of resection (EOR), and residual tumor volume (RV) achieved using ioMRI.

**Methods:**

A systematic review was carried out on PubMed/MEDLINE to identify any studies reporting intra- and postoperative (1) GTR, (2) EOR, or (3) RV in patients who underwent resection of pituitary adenomas with ioMRI. Random effects meta-analysis of the rate of improvement after ioMRI for these three surgical outcomes was intended.

**Results:**

Among 34 included studies (2130 patients), the proportion of patients with conversion to GTR (∆GTR) after ioMRI was 0.19 (95% CI 0.15–0.23). Mean ∆EOR was + 9.07% after ioMRI. Mean ∆RV was 0.784 cm^3^. For endoscopically treated patients, ∆GTR was 0.17 (95% CI 0.09–0.25), while microscopic ∆GTR was 0.19 (95% CI 0.15–0.23). Low-field ioMRI studies demonstrated a ∆GTR of 0.19 (95% CI 0.11–0.28), while high-field and ultra-high-field ioMRI demonstrated a ∆GTR of 0.19 (95% CI 0.15–0.24) and 0.20 (95% CI 0.13–0.28), respectively.

**Conclusions:**

Our meta-analysis demonstrates that around one fifth of patients undergoing pituitary adenoma resection convert from non-GTR to GTR after the use of ioMRI. EOR and RV can also be improved to a certain extent using ioMRI. Endoscopic versus microscopic technique or field strength does not appear to alter the impact of ioMRI. Statistical heterogeneity was high, indicating that the improvement in surgical results due to ioMRI varies considerably by center.

**Supplementary Information:**

The online version contains supplementary material available at 10.1007/s11102-021-01147-2.

## Introduction

Pituitary adenomas (PA) are among the most common intracranial neoplasms, an can become symptomatic due to endocrine and mass effect manifestations [1]. Transsphenoidal surgery (TSS), either endoscopic or microsurgical, represents the standard of care for those patients requiring treatment, except for prolactinomas which can often be managed medically [2–5]. In most patients, gross total resection (GTR) is the surgical goal and can be achieved in the majority of patients [2, 6]. The likelihood of GTR is determined by several factors, including—but not limited to—PA diameter and volume, sellar and dural anatomy, histological features, cavernous sinus invasion, as well as surgical strategy [2, 7–10]. Classifications have been developed for pre-operative evaluation of resectability, such as the Knosp classification and the Zurich pituitary score [7, 9, 11, 12].

In order to increase the proportion of patients where an optimal surgical resection is achieved, a number of studies evaluated the implementation of intra-operative MRI (ioMRI) which, in principle, allows to more accurately identify tumor remnants and has been extensively reported to improve surgical result, independently of surgical strategy [11, 13–19]. This in turn reduces the need for revision surgery, which has a higher inherent risk compared to primary interventions [20, 21]. Although several authors have reported their experience with low-field, high-field, and ultra-high field ioMRI, there is currently a lack of sufficiently powered studies to assess the real benefits in terms of surgical outcomes (GTR, extent of resection [EOR], and residual tumor volume [RV]). [15, 16, 18, 22, 23] Moreover, controversies exist in the literature on the impact of ioMRI in TSS, whether endoscopic or microsurgical [15, 17, 22]. Concerns over false positive and false negative findings, as well as excessive costs—and resulting limited availability—and increased surgical time have been raised, and warrant additional scrutiny [24].

Systematic reviews and statistical meta-analyses can lead to more realistic results through incorporation of data from many centers and consequently many surgeons, as well as increasing statistical power compared to single studies. We systematically reviewed the literature to evaluate the impact of low-, high- and ultra-high field ioMRI on GTR, EOR, and RV in endoscopic or microscopic transsphenoidal pituitary adenoma surgery.

## Materials and methods

### Overview

A systematic review was carried out to identify any studies reporting intra- and postoperative (1) GTR (rate of radiological gross total resection), (2) EOR (proportion of resected tumor volume compared to the preoperative tumor volume), or (3) RV (residual tumor volume in cm^3)^ in patients who underwent resection of pituitary adenomas with ioMRI guidance. Title and abstract screening, full-text review, and data extraction were handled independently by two reviewers (VES and ATP), and disagreements at any stage were resolved by discussion and consensus. Persisting disagreements were resolved by discussion with a third reviewer (CS). We followed the preferred reporting items for systematic reviews and meta-analyses (PRISMA) protocol [25]. This review was registered on PROSPERO (www.crd.york.ac.uk/prospero, Record ID: 177126).

### Search strategy

The PubMed / MEDLINE database was searched to identify eligible articles. The search strategy included combinations of the following terms: pituitary; intraoperative MRI; magnetic resonance imaging; intraoperative; intraoperative imaging; MRI; gross total resection; GTR; extent of resection; EOR; residual; and residual volume (see Table, Supplementary material 1). Word variations and exploded medical subject headings were searched for whenever feasible. Additionally, reference lists were hand-searched to identify further studies of interest. The last comprehensive search was conducted on March 16th 2020.

### Study selection

Only in vivo studies enrolling humans of all age groups in English, Italian, French, Dutch, and German were considered. As a small number of controlled trials were anticipated, prospective and retrospective single-arm cohort studies and case series of adult and pediatric individuals were also included. Case reports and small case series with less than 5 patients were excluded. To be considered, patients had to undergo endoscopic or microscopic trans-sphenoidal resection of pituitary adenomas using ioMRI. Studies had to assess at least one of the three abovementioned outcomes of interest at a minimum of the intraoperative and postoperative time points. In this way, we were able to rate the potential improvement in resection achieved after intraoperative imaging. Studies reporting only resection of Rathke cleft cysts, craniopharyngiomas, or other lesions were excluded. We also excluded studies dealing mainly with transcranial or combined procedures. Studies dealing primarily with patients in which decompression only was the surgical target were excluded. Studies reporting the outcomes of interest with a mix of targeted GTR and STR (i.e. a realistic caseload) were included. Exact cohort duplicates were excluded, although we did include updates of previously published cohorts with a sample size increase of at least 50%.

### Data extraction and quality assessment

We extracted the following information if available from all included publications: study design and year of publication, number of patients, mean patient age and gender distribution, endoscopic or microscopic surgery, low-field (< 1.5 T), high-field (≥ 1.5 T), or “ultra-high” field (≥ 3 T) ioMRI [26], as well as intra- and postoperatively at least one of (1) GTR, (2) EOR, (3) RV. We also assessed whether studies evaluated primarily primary adenoma resections, or primarily revision surgeries. If exclusively reported separately, we included the outcome measures for those patients with targeted GTR. Methodological quality of included studies was graded using the newcastle–ottawa quality assessment scale for cohort studies [27].

### Statistical meta-analysis

The methodology for statistical meta-analysis of related samples is controversial and not well-established. As we were interested in the effect size of ioMRI on GTR, EOR, and RV before vs. after ioMRI, we calculated the individual differences (before vs. after ioMRI) in these three outcomes per study. These effect sizes were then meta-analyzed, if enough appropriate data was available. Because major heterogeneity among the studies in terms of demographics, surgical techniques, and so forth was expected, a random effects meta-analysis was decided upon. ∆GTR was meta-analyzed using the generic inverse variance method, with a Freeman-Tukey Double arcsine transformation to estimate overall proportions [28]. A formal meta-analysis of ∆EOR and ∆RV was not possible as most studies reporting these data did not provide measures of variance (i.e. standard deviations). For this reason, we were only able to calculate patient-weighted means for ∆EOR and ∆RV [29]. We performed stratified analyses for endoscopic and microscopic surgery, as well as for low versus high field ioMRI. Additionally, we evaluated the effect of ioMRI in the “ultra-high” field cohorts (≥ 3 T) [26]. All statistical analyses were carried out in R using the “meta” package [30]. Forest plots were generated to illustrate the main results of the meta-analysis.

## Results

### Literature search

The PubMed/MEDLINE search yielded 432 articles to which an additional 5 were added after retrieval from other sources. A PRISMA flowchart is shown in Supplementary Fig. 1. After duplicate removal (n = 1), 436 records were screened, and 58 were assessed for eligibility through full-text screening. Of the 34 publications included for qualitative synthesis, all were also eligible for quantitative meta-analysis.[11, 13, 16–20, 22–24, 31–55]

### Included study characteristics

Overview of the characteristics of the included studies is reported in Table 1. We identified 12 studies reporting use of low-field ioMRI. Sixteen studies used high-field ioMRI, six studies used ultra-high field ioMRI. With respect to surgical technique, 14 studies used an endoscopic resection technique while 19 studies used the microscopic technique. All included studies evaluated intra-operative and post-operative GTR rates, allowing the calculation of ∆GTR after ioMRI. Only 2 studies reported EOR improvement granted by use of ioMRI [11, 17], and only 4 assessed RV change after ioMRI [11, 17, 39, 44]. (Table 2).

**Table 1:** Overview of the characteristics of the 34 included studies

Author	Year	No. pts	Microscopic/Endoscopic, n	Field strength	NFPA, n (%)	Age, mean (± SD or range)	Male, n (%)	Newcastle–Ottawa scale (S/C/O)
Low-field								
Ahn et al	2008	51	51/0	0.15 T Polestar N20	NA	NA	NA	3/0/3
Berkmann et al	2012	115	115/0	0.15 T Polestar N20	79 (69)	NA	NA	3/0/3
Bohinski et al	2001	29	29/0	0.3 T AIRIS II	22 (76)	51 (24–74)	18 (62)	3/0/3
Garcia et al	2017	30	0/30	0.15 T Polestar N30	15 (50)	55	13 (43)	3/0/3
Hlavica et al	2013	104	104/0	0.15 T Polestar N20	104 (100)	59 (22–86)	57 (55)	3/0/3
Jimenez et al	2016	18	0/18	0.15 T Polestar N20	10 (56)	NA	NA	3/0/3
Martin et al	1999	5	5/0	0.5 T	0 (0)	36.2 (28–42)	2 (40)	3/0/3
Ramm-Pettersen et al	2011	20	20/0	0.5 T Signa SP	16 (80)	54 (23–71)	13 (65)	3/0/3
Schwartz et al	2006	15	0/15	0.12 T Polestar N10	11 (73)	49 (29–67)	9 (60)	3/0/3
Steinmeier et al	1998	18	18/0	0.2 T	15 (83)	21–79	9 (50)	3/0/3
Strange et al	2019	231	0/231	0.15 T Polestar N20	160 (69)	55.5 (18–88)	127 (55)	3/0/3
Wu et al	2009	55	55/0	0.15 T Polestar N20	29 (53)	45.9 (± 12.6)	36 (65)	3/0/3
High-field								
Berkmann et al	2014	85	85/0	1.5 T Magnetom	85 (100)	55 (± 14)	57 (67)	3/0/3
Chen et al	2012	13	13/0	1.5 T Magnetom	NA	NA	NA	3/0/3
Dort et al	2001	15	15/0	1.5 T	NA	50 (15–80)	8 (53)	3/0/3
Gohla et al	2019	42	42/0	1.5 T Espree	35 (83)	52 (17–79)	23 (55)	3/0/3
Hlavac et al	2019	111	66/45	1.5 T Espree	91 (82)	57.3 (22–78)	75 (68)	3/0/3
Kuge et al	2013	35	0/35	1.5 T	27 (77)	54.3 (± 15.5)	18 (51)	3/0/3
Li et al	2015	30	30/0	1.5 T Espree	9 (30)	36 (21–65)	13 (43)	3/0/3
Nimsky et al	2004	48	48/0	1.5 T	NA	NA	NA	3/0/3
Nimsky et al	2006	85	85/0	1.5 T Magnetom	85 (100)	NA	NA	3/0/3
Pal’a et al	2017	96	68/28	1.5 T Espree	64 (67)	54 (7–78)	71 (74)	3/0/3
Paterno et al	2014	49	0/49	1.5 T Espree	49 (100)	NA	NA	
Sylvester et al	2015	156	115/41	1.5 T Espree	NA	NA	NA	3/0/3
Szerlip et al	2011	53	53/0	1.5 T Espree	39 (74)	49 (1.8 SEM)	25 (47)	3/0/3
Tanei et al	2013	14	0/14	1.5 T Magnetom	0 (0)	37.4 (± 11.8)	2 (14)	3/0/3
Zhang et al	2017	137	0/137	1.5 T Espree	103 (75)	7–82	73 (53)	
Zhang et al	2019	133	0/133	1.5 T Espree	133 (100)	50 (± 12)	61 (46)	3/0/3
Ultra-high-field								
Fomekong et al	2014	73	73/0	3 T Intera	NA	50 (17–84)	46 (63)	3/0/3
Netuka et al	2011	49	NA	3 T	NA	NA	NA	3/0/3
Qiu et al	2012	49	NA	3 T Mangetom	NA	NA	NA	3/0/3
Serra et al	2016	51	0/51	3 T Mangetom	33 (65)	52 (21–83)	27 (53)	3/0/3
Staartjes et al	2019	95	0/95	3 T Magnetom	65 (68)	53.8 (20–82)	53 (56)	3/0/3
Zaidi et al	2016	20	0/20	3 T Verio	14 (70)	51.6 (34–72)	9 (45)	3/0/3

*NFPA* non-functioning pituitary adenoma, *SD* standard deviation, *NA* not applicable

**Table 2:** Data on gross total resection, extent of resection, and residual tumor volumes extracted from the 34 included studies

Author	Year	GTR (%) (ioMRI)	GTR (%) (postop)	ΔGTR (%)	EOR (%) (ioMRI)	EOR (%) (postop)	ΔEOR (%)	RV (cm^3^) (ioMRI)	RV (cm^3^) (postop)	ΔRV (cm^3^)
Low-field										
Ahn et al	2008	74.5	94.1	19.6						
Berkmann et al	2012	61.0	82.0	21.0						
Bohinski et al	2001	24.1	55.2	31.1						
Garcia et al	2017	63.3	83.3	20.0						
Hlavica et al	2013	46.2	67.3	21.1						
Jimenez et al	2016	44.4	77.8	33.3						
Martin et al	1999	40.0	80.0	40.0						
Ramm-Pettersen et al	2011	40.0	60.0	20.0						
Schwartz et al	2006	80.0	86.6	6.6						
Steinmeier et al	1998	–	–	16.7						
Strange et al	2019	48.0	52.0	4.0						
Wu et al	2009	58.2	83.6	25.4						
High-field										
Berkmann et al	2014	44.0	66.0	22.0						0.900 (1.7)
Chen et al	2012	38.5	76.9	38.4						
Dort et al	2001	73.3	93.3	20.0						
Gohla et al	2019	28.6	42.9	14.3						
Hlavac et al	2019	29.7/25.8/35.6	39.4/36.4/44.2	9.7/10.6/8.6				2.13/2.445/1.642	1.199/1.220/1.165	0.939/1.225/0.477
Kuge et al	2013	65.7	71.4	5.7						
Li et al	2015	60.0	80.0	20.0						
Nimsky et al	2004	56.2	87.5	31.3						
Nimsky et al	2006	58.0	82.0	24.0						
Pal’a et al	2017	47.9 /42.6/60.7	60.4/55.9/72.4	12.5/13.3/11.7	77.7/74.0/87.4	89.7/87.9/95.3	12.0/13.9/7.9	1.752/2.137/0.873	0.810/0.994/0.329	0.942/1.143/0.544
Paterno et al	2014	47.0	100	53.0						
Sylvester et al	2015	28.2	35.9	7.7						
Szerlip et al	2011	37.7	62.3	24.6						
Tanei et al	2013	50.0	78.6	28.6						
Zhang et al	2017	67.2	81.0	13.9						
Zhang et al	2019	42.9	63.9	21.0						
Ultra-high-field										
Fomekong et al	2014	58.9	72.6	13.7						
Netuka et al	2011	69.4	91.8	22.4						
Qiu et al	2012	77.6	85.7	8.2						
Serra et al	2016	31.0	61.0	30.0						
Staartjes et al	2019	44.0	72.0	28.0	92.1 (± 13.3)	98.2 (± 3.8)	6.1	0.47 (± 1.57)	0.13 (± 0.34)	0.34
Zaidi et al	2016	60.0	80.0	20.0						

*GTR* gross total resection, *EOR* extent of resection, *RV* residual tumor volume, *ioMRI* intraoperative MRI

### Gross total resection

**Fig. 1:**
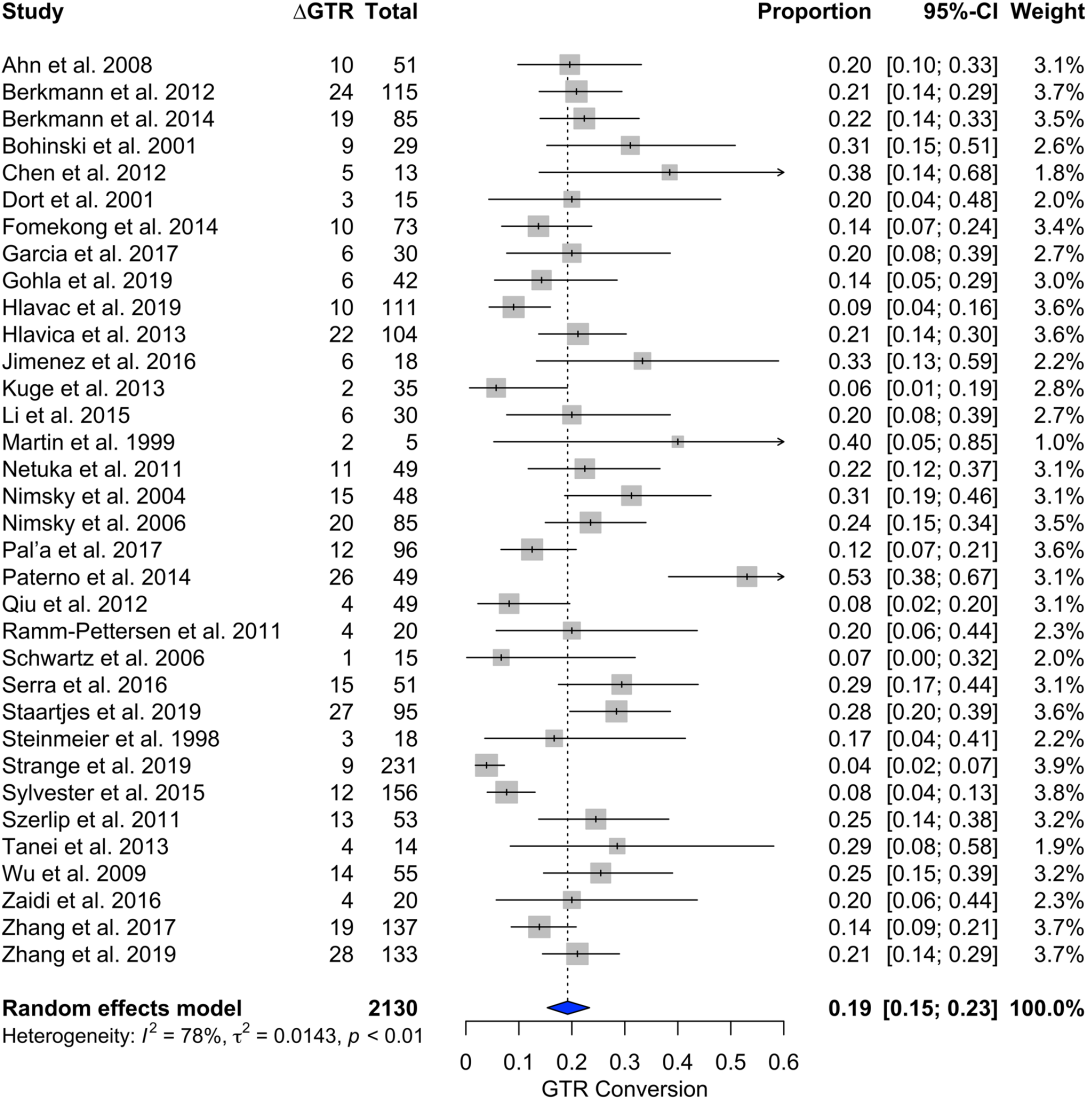
Forest plot representing the results of the statistical meta-analysis of the change in gross total resection (∆GTR) in percent from intraoperative to postoperative imaging

Random-effect meta-analysis showed that, in the 34 included studies (2130 patients), the proportion of patients with conversion to GTR (∆GTR) after ioMRI was 0.19 (95% CI 0.15–0.23). Heterogeneity—as measured by I^2^ statistic—was high with 78% (p < 0.01). (Fig. 1).

Extent of resection and residual tumor volume.

Formal meta-analysis was not possible for EOR and RV, thus patient-weighted means were calculated instead (Table 3). Among 191 patients, ∆EOR was + 9.07% after ioMRI on average.

**Table 3:** Patient-weighted means of the two outcomes not amenable to formal meta-analysis. For residual volume, a subgroup analysis of endo- and microscopic cases was feasible

Parameter	N	Case-weighted mean
ΔEOR (%)		
Overall	191	9.07
ΔRV (cm^3^)		
Overall	387	0.784
Endoscopic	73	0.503
Microscopic	134	1.183

*EOR* extent of resection, *RV* residual volume

Concerning RV, overall ∆RV was 0.784 cm^3^. Subgroup analysis stratified by surgical techniques was possible, with endo- (n = 73) and microscopic patients (n = 134) demonstrating an average ∆RV of 0.503 cm^3^ and 1.183 cm^3^, respectively.

### ioMRI in endoscopic versus microscopic technique

**Fig. 2:**
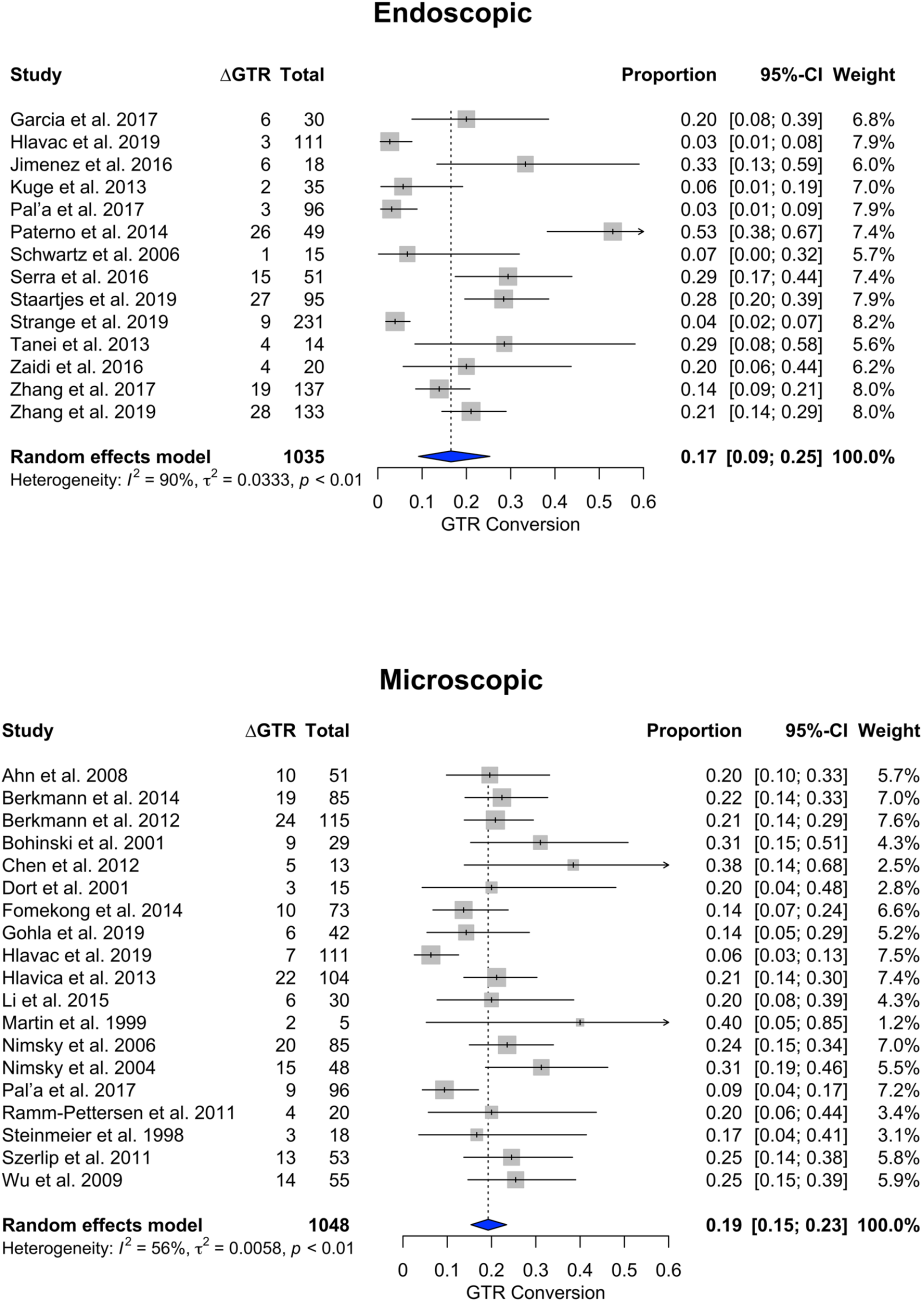
Stratified analysis of endoscopic versus microscopic surgery. Forest plots represent the results of the statistical meta-analysis of the change in gross total resection (∆GTR) in percent from intraoperative to postoperative imaging

When only studies assessing endoscopic surgery (n = 14) were evaluated (1035 patients), ∆GTR proportion was 0.17 (95% CI 0.09–0.25), while in studies performing microscopic TSS (n = 19, 1048 patients), the GTR proportion was 0.19 (95% CI 0.15–0.23) (Fig. 2).

### Low- versus high- versus ultra high-field ioMRI

**Fig. 3:**
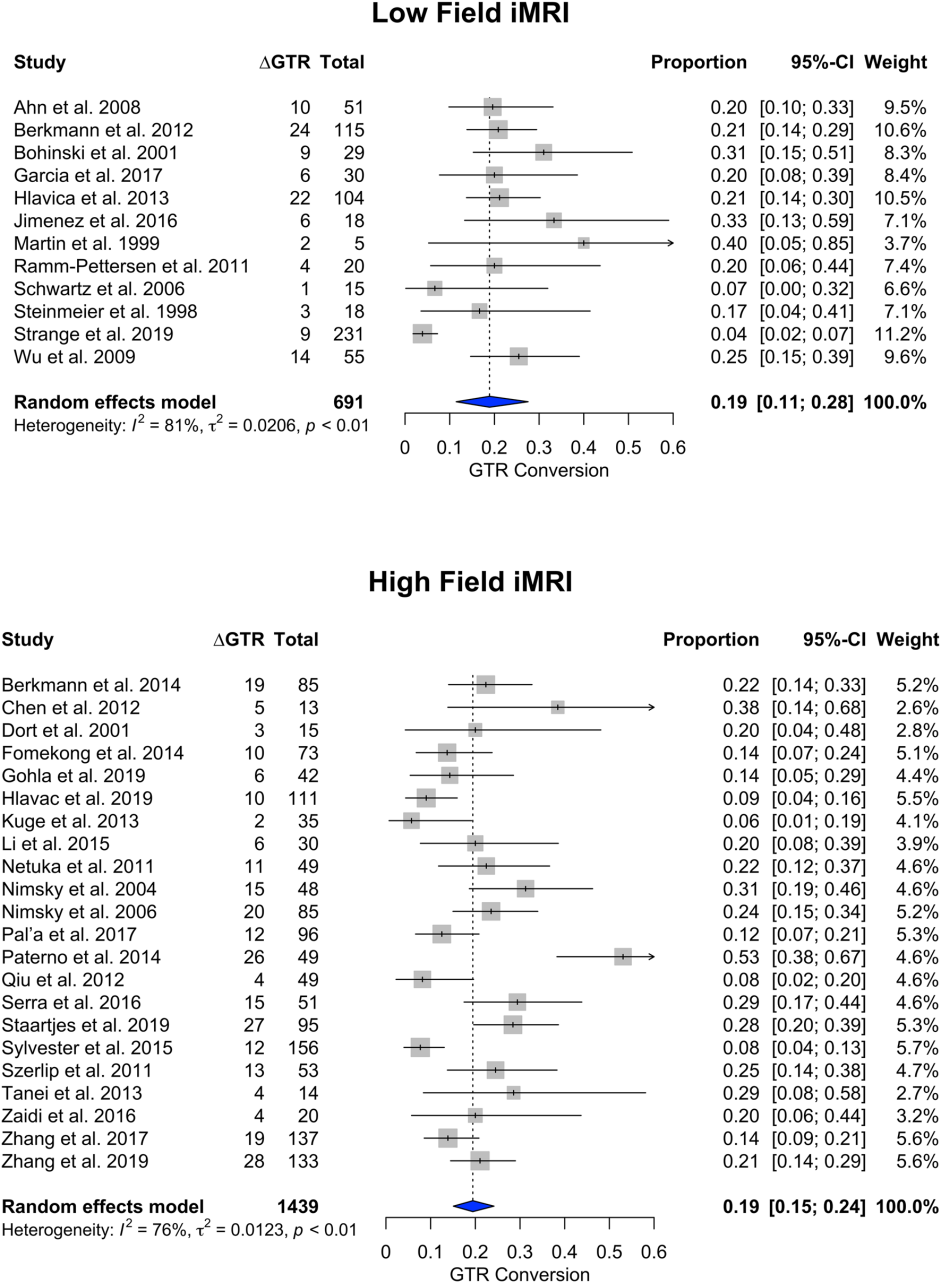
Stratified analysis of low-field versus high-field transsphenoidal surgery. Forest plots represent the results of the statistical meta-analysis of the change in gross total resection (∆GTR) in percent from intraoperative to postoperative imaging

**Fig. 4:**
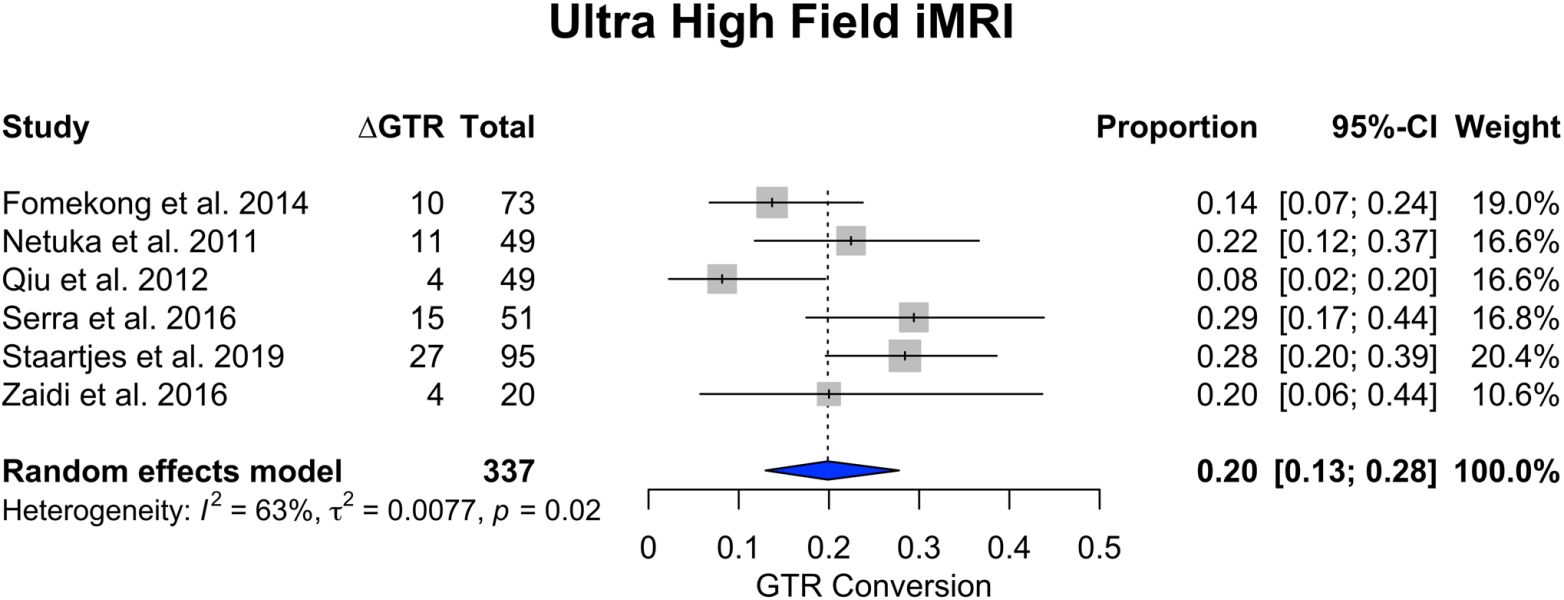
Forest plot of studies including ultra high-field intraoperative MRI, representing the results of the statistical meta-analysis of the change in gross total resection (∆GTR) in percent from intraoperative to postoperative imaging

Low-field ioMRI studies (n = 12) accounting for 691 patients demonstrated a ∆GTR proportion of 0.19 (95% CI 0.11–0.28), while meta-analysis (Fig. 3) of publications reporting high-field ioMRI (n = 16) among 1439 patients had a ∆GTR proportion of 0.19 (95% CI 0.15–0.24). When studies employing ultra high-field ioMRI (Fig. 4) were meta-analyzed, (n = 6) the ∆GTR proportion was 0.20 (95% CI 0.13–0.28) among 337 patients.

## Discussion

Our meta-analysis demonstrated that the use of ioMRI—on average—grants an additional conversion to GTR in PA patients undergoing surgery ranging between 15 and 23%. EOR increased by an average of 9%, and RV decreased by 0.784 cm^3^. In addition, when evaluating studies assessing ioMRI benefit in endoscopic TSS versus microscopic surgery separately, ∆GTR was similar. When comparing different magnetic field strengths, no major statistically significant differences could be accounted for.

Regarding the benefit of ioMRI in either microscopic or endoscopic surgery, a recent study by Soneru et al. compared literature data on GTR after pituitary adenoma resection, and found endoscopic TSS with or with- out ioMRI resulted in a similar rate of GTR when compared to patients undergoing ioMRI-assisted microscopic TSS for all pituitary adenomas [15]. For macroadenomas, the pooled rate of GTR in endoscopic TSS + ioMRI was higher than microscopic TSS + ioMRI. More importantly, Soneru et al. found that ioMRI lead to a higher rate of GTR than endoscopic TSS alone, even if comparisons were indirect [15, 55]. Their results, however have to be interpreted cautiously due to great heterogeneity among the included studies, which could not be corrected by meta-regression [2, 15]. It is important to consider that the contributions of ioMRI to GTR conversion are thought to derive mainly from identification of additional intrasellar remnants which are prone to safe surgical resection [13]. Additional advantages which may result from ioMRI may include increased EOR and therefore decreased RV and early identification of complications [16, 52, 53]. There is even some weak evidence that early post-operative imaging correlates more poorly with long-term surgical outcome than intra-operative imaging [16].

Thus, focusing on GTR only may underestimate the resection improvement granted by ioMRI. A limited number of studies assessed EOR and RV improvement due to ioMRI. Therefore, we were unable to carry out statistical meta-analysis, although our numbers still show a small improvement in EOR and RV. Given the low number of studies and limited sample size, future studies should evaluate this question with appropriate design and methodology, including precise volumetric tumor remnant segmentation [13, 17]. The improvement granted by increased extent of resection has been shown to positively influence progression free survival (PFS), and may also make the tumor remnant more amenable to salvage treatments such as radiosurgery or, more importantly, to spare the patient from revision surgery, which is intrinsically associated with increased risks [37, 49]. Some authors also suggest that tumor remnants visualized using ioMRI in a significant number of patients may not be amenable to further safe resection, and that when aggressive resection is pursued, this may in turn lead to increased complications such as cerebrospinal fluid leak, arterial injury and hypopituitarism—even if the literature seem to rule out such occurrences [56].

In our study, we were unable to identify any selective advantage of high- or even ultra high-field compared to low-field ioMRI. This conclusion must be taken cautiously since we could not ascertain if the different patients cohorts were comparable concerning baseline variables known to affect the likelihood of achieving GTR. Published case series suggested that low-field ioMRI already improved GTR [18, 19, 37, 55, 57]. Potential explanations for this finding are that low-field MRI already provides visualization accurate enough to identify remnants amenable to further resection, but that those remnants that cannot be resected after either high- or low-field ioMRI are not amenable to resection, either way. For example, tumor remnants lateral to the carotids or invading the cavernous sinus profoundly may not be resettable even if detected at ioMRI.—There is however not enough data to assess if high- or ultrahigh field may provide better EOR and RV rates, being both outcomes particularly valuable in secreting adenomas. The evidence on this topic is controversial, as some authors report absence of false positive but variable false negative findings with low-field, but not with high-field ioMRI [18, 23]. The perceived improvement may be more relevant in patients with functioning PAs, where size of the residual volume is more closely linked with endocrine remission. It has been claimed that high-field ioMRI can possibly grant increased sensitivity in patients of subtotal resection specifically in microadenomas [37, 58]. At the same time, parasellar anatomy, cavernous sinus invasion and small lesions cannot be as reliably evaluated—according to some authors—with low-field than with high-field ioMRI [19].

The cost-benefit ratio favors ioMRI use, even when increased costs are accounted for as the increased rate of GTR reduces reoperations or additional therapies and their associated expenses, according to a recent analysis [55]. Limiting the use of such technology to patients where the benefit is clearer such as those with suprasellar extension has been suggested as a viable strategy to further reduce costs [20]. Predictive tools such as the Zurich pituitary score, which has demonstrated its ability to predict in which patients ioMRI may be most useful, could be used for cost–benefit assessment [9, 11, 12]. When applying the Zurich pituitary score, it has been found that ioMRI is most useful in Grade I and II patients—small tumors—where GTR can almost always be achieved in a safe fashion when ioMRI is applied, compared to Grade IV adenomas which are seldom amenable to GTR anyway—In these patients, ioMRI can serve to increase EOR.

Past literature correctly points out at the main concern in evaluating ioMRI results, namely that knowledge of ioMRI availability may result in a more conservative first resection, falsely increases the conversion rate enabled by ioMRI [13, 16, 24, 48, 55]. Randomized studies comparing ioMRI to no-ioMRI are not available and none are ongoing. Some authors report that intra-operative imaging was pursued only when the neurosurgeon believed GTR had been achieved or when additional potentially unnecessary exploration was feared due to the risk of complications or morbidity [52]. Important concerns remain related to selection bias, lack of blinding in the evaluation of the resection, and a lack of randomized studies. Irrespective of this bias, reports about the early intraoperative identification of complications and proven advantages such as increasing maximally safe resection add to the evidence supporting the use of ioMRI [20]. Future studies evaluating the use of ioMRI should ideally assess not only GTR but also EOR an RV quantitatively to better evaluate its contribution and to allow formal meta-analysis [13].

### Limitations

The main limitation is that there are no data stemming from randomized studies. This only allows us to describe the real-world improvements in GTR, EOR, and RV observed, without considering the implicit biases described above. Due to the substantial heterogeneity observed, our results have to be interpreted with some caution and suggest a large variability in the use and consequences of ioMRI in different centers. Intrinsic biases of included publications cannot be ruled out. EOR and RV were evaluated using very limited data from only few studies. Because of a lack of granularity in the data identified in our systematic review, we were unable to perform stratification for functioning versus non-functioning adenoma. Additionally, we did not include endocrinological remission as an outcome of interest. Tumor size stratification was not possible, limiting our insights on the benefit of ioMRI for small versus large adenomas. Other outcomes such as safety and cost-effectiveness were not investigated.

## Conclusion

Our meta-analysis demonstrates that around one fifth of patients undergoing pituitary adenoma resection convert from non-GTR to GTR after the use of ioMRI, in accordance with previous findings. EOR and RV can also be improved to a certain extent using ioMRI. When considering GTR, the benefit of ioMRI does not change for endoscopic versus microscopic transsphenoidal surgery, nor does field strength seem to influence results. Statistical heterogeneity was high, indicating that the improvement in surgical results due to ioMRI varies considerably by center. While it is likely that ioMRI truly increases GTR and EOR and leads to lower RV, only randomized studies can take this question to a higher level of evidence by avoiding the implicit biases introduced through the mere use of ioMRI. Regardless, future studies on ioMRI should provide quantitative assessment of surgical results, including volumetric assessment of EOR and RV.

## Declarations

## Conflict of interest

The authors declare that the article and its content were composed in the absence of any commercial or financial relationships that could be construed as a potential conflict of interest.

## Supplementary Information

Below is the link to the electronic supplementary material.

Supplementary file1 (TIF 2405 KB)
