# Supplementary figures and images for: Impact of intraoperative magnetic resonance imaging on gross total resection, extent of resection, and residual tumor volume in pituitary surgery: systematic review and meta-analysis
Source: Pituitary. 2021 May 4;24(4):644–56. doi: 10.1007/s11102-021-01147-2 (PMC8270798; doi:10.1007/s11102-021-01147-2)

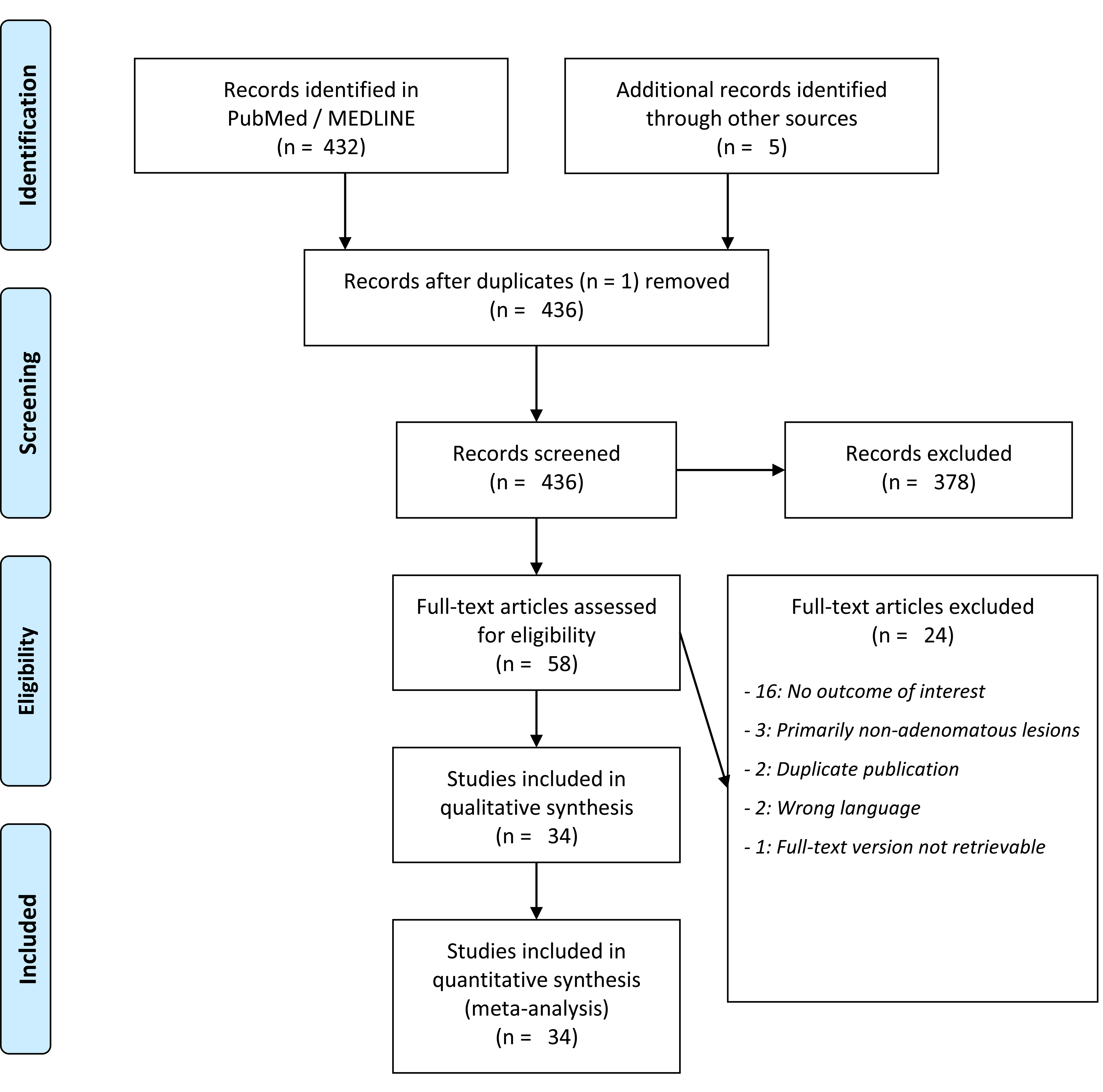

Supplement: Supplementary file 1 — Supplementary file1 (TIF 2405 KB) [file 11102_2021_1147_MOESM1_ESM.tif]
